# Potential of genotyping-by-sequencing for genomic selection in livestock populations

**DOI:** 10.1186/s12711-015-0102-z

**Published:** 2015-03-01

**Authors:** Gregor Gorjanc, Matthew A Cleveland, Ross D Houston, John M Hickey

**Affiliations:** The Roslin Institute and Royal (Dick) School of Veterinary Studies, The University of Edinburgh, Easter Bush, Midlothian, Scotland UK; Genus Plc, 100 Bluegrass Commons Blvd., Suite 2200, Hendersonville, TN 37075 USA

## Abstract

**Background:**

Next-generation sequencing techniques, such as genotyping-by-sequencing (GBS), provide alternatives to single nucleotide polymorphism (SNP) arrays. The aim of this work was to evaluate the potential of GBS compared to SNP array genotyping for genomic selection in livestock populations.

**Methods:**

The value of GBS was quantified by simulation analyses in which three parameters were varied: (i) genome-wide sequence read depth (*x*) per individual from 0.01*x* to 20*x* or using SNP array genotyping; (ii) number of genotyped markers from 3000 to 300 000; and (iii) size of training and prediction sets from 500 to 50 000 individuals. The latter was achieved by distributing the total available *x* of 1000*x*, 5000*x*, or 10 000*x* per genotyped locus among the varying number of individuals. With SNP arrays, genotypes were called from sequence data directly. With GBS, genotypes were called from sequence reads that varied between loci and individuals according to a Poisson distribution with mean equal to *x*. Simulated data were analyzed with ridge regression and the accuracy and bias of genomic predictions and response to selection were quantified under the different scenarios.

**Results:**

Accuracies of genomic predictions using GBS data or SNP array data were comparable when large numbers of markers were used and *x* per individual was ~1*x* or higher. The bias of genomic predictions was very high at a very low *x*. When the total available *x* was distributed among the training individuals, the accuracy of prediction was maximized when a large number of individuals was used that had GBS data with low *x* for a large number of markers. Similarly, response to selection was maximized under the same conditions due to increasing both accuracy and selection intensity.

**Conclusions:**

GBS offers great potential for developing genomic selection in livestock populations because it makes it possible to cover large fractions of the genome and to vary the sequence read depth per individual. Thus, the accuracy of predictions is improved by increasing the size of training populations and the intensity of selection is increased by genotyping a larger number of selection candidates.

**Electronic supplementary material:**

The online version of this article (doi:10.1186/s12711-015-0102-z) contains supplementary material, which is available to authorized users.

## Background

Current applications of genomic selection (GS) in livestock are typically based on single nucleotide polymorphism (SNP) genotypes called from SNP array data. In practice, combinations of high and low-density SNP arrays along with imputation are used to reduce the costs of genotyping [1-3]. These low-cost genotyping strategies enable increased intensity of selection through the genotyping of large numbers of selection candidates or increased accuracy of estimated breeding values (EBV) by expanding the training population. If datasets of both phenotyped and genotyped individuals (the training population) become very large, the predictive power of GS may be increasingly driven by linkage disequilibrium rather than by linkage information, or, more usefully, by direct genotyping of causative mutations and other biologically relevant genomic information [4,5]. Thus, this may provide an opportunity to increase the power of GS in breeding programs; however, to fully capitalize on this potential it is necessary to genotype larger numbers of individuals for a greater fraction of the genome than what is typically covered by current SNP arrays.

Use of next-generation sequencing (NGS) techniques for genotyping could be a viable alternative to current low-cost SNP array strategies, with the potential to increase the fraction of the genome captured in a cost-efficient manner [6-8]. Genotyping-by-sequencing (GBS) uses NGS technology to genotype large numbers of individuals and has the potential to drive the cost per sample below $10 through intensive multiplexing [9]. It has been applied with success in plants [7,10] and cattle [11]. GBS and similar techniques such as RAD-Seq [6] are reduced representation approaches that use restriction enzymes to target the sequencing effort to a fraction of the genome. This fraction of the genome can be readily adjusted according to the needs of the project and can potentially be much greater than the fraction captured by even the densest SNP arrays currently available in livestock. Furthermore, unlike SNP arrays that are typically developed from a limited sample of individuals, GBS can capture genetic variation that is specific to a population or family of interest, e.g., [11-14]. However, compared to genotypes obtained from SNP arrays, the quality of genotypes obtained with GBS tends to be lower since it depends on the genome-wide sequence read depth (*x*). By increasing *x*, the proportion of correctly called genotypes increases but so do the costs. Since *x* varies along each sequenced genome, the number and quality of genotype calls also vary along the genome of each individual [15-17]. These drawbacks complicate the use of GBS data, but can be partially overcome by imputation and error correction methods [18-20].

GBS has been shown to be useful for GS of advanced breeding lines of wheat [9] and of double haploid or inbred lines of maize [20]. In these applications, read depth as low as ~1*x* was sufficient to obtain accurate EBV without using imputation and error correction methods. This was in part facilitated by the low levels of heterozygosity in the individuals analyzed in these studies. However, the usefulness of GBS for GS in outbred livestock populations with higher levels of heterozygosity has not yet been evaluated. Algorithms and software for imputation and error correction of NGS data in livestock have yet to be developed to capitalize on their unique population structure and the available information (e.g., pedigree information, large family sizes and close relatives). Therefore, for GBS to be a viable alternative to current genotyping approaches with SNP arrays in livestock, it needs to be competitive in the absence of imputation and error correction methods.

The objective of this research was to quantify the potential of GBS for GS in outbred populations of livestock in the absence of imputation and error correction methods. Specifically, using simulations, the accuracy and bias of predictions and response to selection were compared for various genome-wide sequence read depths (*x*) and fractions of genome covered. The results show that the accuracies of EBV obtained with non-imputed GBS data and of SNP array data were comparable when *x* was as low as ~1*x* and large fractions of genome were covered. In addition, decreasing *x* per individual enabled an increase in the response to selection by increasing both accuracy of prediction and intensity of selection through exploitation of the trade-off between the quality of genotyping and the number of individuals that could be genotyped.

## Methods

To test the usefulness of GBS data for GS, the effects of genome-wide sequence read depth, fraction of the genome covered, and the size of the training and prediction sets were quantified using simulation with ten replicates. The results were represented with a mean over replicates. In addition, individual replicates were presented to indicate variability of results where feasible. In summary, the simulations consisted of four steps to generate: (i) data on whole-genome sequence; (ii) the pedigree structure for a livestock population; (iii) causative loci affecting phenotypes; and (iv) marker genotypes. These simulated data were in turn used in the analyses described below.

### Sequence

Sequence data were generated using the Markovian Coalescent Simulator (MaCS) [21] and AlphaDrop [22] for 1000 base haplotypes for each of 30 chromosomes. Each chromosome was 100 cM long and included 10^8^ base pairs. Chromosomes were simulated using a per site mutation rate of 2.5 × 10^−8^, a per site recombination rate of 1.0 × 10^−8^, and an effective population size (*N*_*e*_) that varied over time. Based on estimates for the Holstein cattle population [23], effective population size was set to *N*_*e*_ = 100 in the final generation of simulation, to *N*_*e*_ = 1256 for 1000 years ago, to *N*_*e*_ = 4350 for 10 000 years ago, and to *N*_*e*_ = 43 500 for 100 000 years ago, with linear changes in between. The resulting sequences had approximately 1.7 million segregating sites in total.

### Pedigree

After the sequence simulation, several pedigrees of two generations were simulated. Chromosomes of individuals in the first generation were sampled from the 1000 simulated base haplotypes and those in the second generation were sampled from the parents’ chromosomes by recombination (crossovers occurred with 1% probability per cM and were uniformly distributed along the chromosomes). Different pedigrees were simulated by mating each of the 25 sires with 20 dams (500 dams in total), with 500, 1000, 5000, 10 000, 20 000, or 50 000 progeny per generation by varying the number of progeny per mating.

### Quantitative trait loci and phenotypes

Quantitative trait loci (QTL) were selected as a sample of 9000 segregating sites in the base population, with the restriction that 300 were sampled from each chromosome. These QTL had their allele substitution effect sampled from a normal distribution with a mean of 0 and standard deviation of 1.0 divided by the square root of the number of QTL. QTL and their effects were in turn used to compute true (simulated) breeding values to simulate complex trait phenotypes with a heritability of 0.25.

### Marker genotypes

The fraction of genome covered by the different genotyping platforms was represented by a variable number of markers, which was selected as a random sample of 3000 (3 K), 9990 (10 K), 60 000 (60 K), and 300 000 (300 K) segregating sites in the base population, with the restriction that equal numbers were sampled from each chromosome. These markers were assumed to be available for all individuals and their genotypes were called via processes similar to those used for either SNP arrays or GBS. With SNP arrays, genotypes were called from sequence data directly without error. With GBS, genotypes were called from sequence data based on the principle and simulation procedure described in the following.

In the absence of sequencing errors, a single sequence read of a locus provides discriminative calls for homozygous but not heterozygous genotypes, which can be called only when multiple sequence reads are available. Calling a heterozygous genotype in diploids from *n* sequence reads is the same as observing two different outcomes among *n* draws from a Bernoulli distribution. Such an event has a probability of 1-2/2^*n*^. The probability of calling a heterozygous genotype from *n* sequence reads of a locus is therefore equal to 0.00 for *n* = 1, 0.50 for *n* = 2, 0.75 for *n* = 3, 0.875 for *n* = 4, etc. However, the number of sequence reads per locus varies along the genome, thus, for an (average) genome-wide sequence read depth (*x*), the realized number of sequence reads per locus *i* of an individual *j* (*n*_*i,j*_) was assumed to be distributed according to a Poisson distribution with mean equal to *x*, i.e., ${n}_{i,j}\sim Poisson(x)$.

GBS genotypes were called from sequencing data with the following range of *x* across the sequenced fraction of the genome: 0.01*x*, 0.02*x*, 0.05*x*, 0.10*x*, 0.20*x*, 0.25*x*, 0.50*x*, 1.00*x*, 1.50*x*, 2.00*x*, 3.00*x*, 4.00*x*, 5.00*x*, 10.00*x*, and 20.00*x*. These values represent the average number of sequence reads at genotyped loci per individual and is often referred also as “coverage” in the literature, e.g., [18]. If one or more reads occurred at a homozygous locus, the correct homozygous genotype was called, and if one or more reads occurred at a heterozygous locus, the heterozygous genotype was at random called with a probability of 1-2/2^*n*^ and one of the homozygous genotypes with a probability of 1/2^*n*^. For example, with two reads at a heterozygous locus, the probability to call each of the homozygous genotypes was 0.25 and the probability to call the heterozygous genotype was 0.50. Uncertainty in the calling of genotypes was neglected, i.e., the collected data were discrete genotype calls and not genotype probabilities or derived allele dosages. It was assumed that all selected SNP sites could be sequenced in all individuals and that sequencing errors were absent. If there was no read for an individual at a particular locus, the genotype was set equal to twice the allele frequency of the allele coded as 1. Allele frequencies were assumed known.

### Training and prediction sets

Training and prediction sets were extracted to test the accuracy of EBV using GBS or SNP array genotype data. The training set comprised all individuals in the first generation (500, 1000, 5000, 10 000, 20 000, or 50 000 individuals) that were genotyped and phenotyped. The prediction set comprised a random subset of 500 genotyped individuals from the second generation, with the restriction that all families were equally represented, i.e., an equal number of progeny per dam was sampled.

### Statistical analysis

Statistical analysis was based on the ridge-regression model [24-26], as implemented in the software AlphaBayes2:

$$ {y}_i\sim N\left({\mu}_i,{\sigma}_e^2\right), $$

$$ {\mu}_i=\alpha +{\displaystyle \sum_{j=1}^p}{\beta}_j{x}_{i,j}, $$

$$ {\beta}_j\sim N\left(0,{\sigma}_{\beta}^2\right), $$

where ${y}_i$ is the phenotype value of the *i*–th individual, *α* is the intercept, *β*_*j*_ and *x*_*i,j*_ are the allele substitution effect and genotype call of the *j*-th marker, and ${\sigma}_e^2$ and ${\sigma}_{\beta}^2$ are, respectively, variances of residuals and of allele substitution effects. Values of the variance components used in the simulation were assumed known to minimize sampling variation. Estimates of allele substitution effects $\left(\widehat{\beta_j}\right)$ were used to compute individual EBV as $\left(\widehat{a_i}={\displaystyle {\sum}_{j=1}^p\widehat{\beta_j{X}_{ij}}}\right)$. Accuracy of EBV was calculated as the correlation between the true breeding values (TBV) and the EBV. Bias of EBV was calculated as the regression of TBV on the EBV, where the desired value is 1.0 and values greater than 1.0 (underestimation of EBV) are preferred to values less than 1.0 (overestimation of EBV).

### Design of the analysis

The simulated data were analyzed in several ways to quantify the effect of: (A1) using the same *x* in both training and prediction sets; (A2) using different *x* in training and prediction sets; (A3) reducing *x* to expand the training set; and (A4) reducing *x* to expand the prediction set. For each of these analyses, all marker densities were used to quantify the effect of the fraction of genome covered.

(A1) The effect of using the same *x* in both training and prediction sets was quantified by training the prediction model on the 1000 individuals with phenotype and genotype information from the first generation and predicting EBV of 500 individuals with genotype information that were randomly sampled from the second generation. In this analysis, GBS data with the whole range of *x* and SNP array data were tested (see Subsection “Marker genotypes”).

(A2) The effect of using different *x* in training and prediction sets was quantified by using the same setting as in (A1), except that all combinations of *x* in training and prediction sets were analyzed (i.e., 0.01*x* in the training set and 0.01*x*, 0.02*x*, etc. in the prediction set, etc.).

(A3) The effect of reducing *x* per individual in order to expand the training set was quantified by training the prediction model on different numbers of individuals with phenotype and genotype information in the first generation (500, 1000, 5000, 10 000, 20 000, or 50 000 individuals) to predict EBV of 500 individuals with genotype information that were randomly sampled from the second generation. Three different *x* per individual were used, such that the total available *x* of 1000*x*, 5000*x*, or 10 000*x* was spread across all individuals in the training set and kept constant across all sizes of training sets. More precisely, with the total of 1000*x*, 5000*x*, or 10 000*x* (Table 1), the training set comprised: (i) 500 individuals at 2*x*, 10*x*, or 20*x*; (ii) 1000 individuals at 1*x*, 5*x*, or 10*x*; (iii) 5000 individuals at 0.2*x*, 1*x*, or 2*x*; (iv) 10 000 individuals at 0.1*x*, 0.5*x,* 1*x*; (v) 20 000 individuals at 0.05*x*, 0.25*x*, or 0.5*x*; or (vi) 50 000 individuals at 0.02*x*, 0.1*x*, or 0.2*x*. The prediction set was genotyped either with SNP arrays, to remove confounding with the quality of genotyping, or with GBS with the same *x* as the training set to maintain consistency.

**Table 1:** **Genome-wide sequence read depth (** ***x*** **) per individual in scenarios with a different total available** ***x*** **and varying number of individuals**

	**Number of individuals**					
	**500**	**1000**	**5000**	**10 000**	**20 000**	**50 000**
**Total** ***x***	**Per individual** ***x***					
1000	2	1	0.2	0.1	0.05	0.02
5000	10	5	1.0	0.5	0.25	0.10
10 000	20	10	2.0	1.0	0.50	0.20

(A4) The effect of reducing *x* per individual to expand the prediction set was quantified by calculating the response to selection (in units of standard genetic deviations) using the breeders’ equation, e.g., [27]. It was assumed that 25 males were selected to become sires of the next generation from a prediction set of 500, 1000, 5000, 10 000, 20 000, or 50 000 individuals. These individuals had GBS data with the same *x* as the training set in (A3), such that the total available *x* in the prediction set was equal to 1000*x*, 5000*x*, or 10 000*x* (Table 1), i.e., (i) 500 individuals at 2*x*, 10*x*, or 20*x*; (ii) 1000 individuals at 1*x*, 5*x*, or 10*x*; (iii) 5000 individuals at 0.2*x*, 1*x*, or 2*x*; (iv) 10 000 individuals at 0.1*x*, 0.5*x,* 1*x*; (v) 20 000 individuals at 0.05*x*, 0.25*x*, or 0.5*x*; or (vi) 50 000 individuals at 0.02*x*, 0.1*x*, or 0.2*x*. Response to selection was calculated in two ways based on the accuracies obtained from the various scenarios in (A1) and (A3). Accuracies from the scenarios in (A1) were used when the training set (1000 individuals) had SNP array data and the prediction set had GBS data with the same *x* as training set in (A3). This set of scenarios was chosen to remove confounding with the quality of genotyping in the training set and to maintain consistency. Accuracies from the scenarios in (A3) were used when both the training and prediction sets had the same *x*. This set of scenarios was chosen to show the potential of expanding both the training and prediction sets by reducing *x* per individual.

## Results

**Figure 1:**
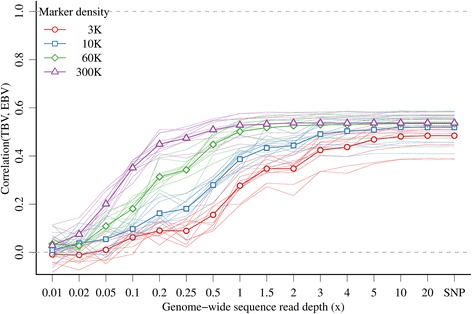
**Accuracy of genomic prediction with GBS data with the same genome-wide sequence read depth or SNP array data in the training and prediction sets and different marker densities.**

(A1) The accuracy of EBV calculated using GBS data was strongly influenced by both *x* and marker density when *x* was the same in the training and prediction sets, as well as by the interaction between these two factors (Figure 1). In general, the accuracy was low at low *x* and increased with increasing *x*. At very low *x* (e.g., 0.01) the accuracy was close to 0 and ranged from −0.01 for 3 K markers to 0.03 for 300 K markers. However, the accuracy increased quickly with small increases in *x*, especially for the higher marker densities. With 300 K markers, the asymptote of accuracy (0.54) was obtained with 2.0*x* and most of this was obtained with 1.0*x* (0.53) or 0.5*x* (0.51). With 60 K markers, the asymptote of accuracy (0.53) was obtained with 3.0*x* and most of this was obtained with 2.0*x* (0.52) or 1.0*x* (0.50). With less than 60 K markers, the asymptote was reached at higher *x*, i.e. 5.0*x* for 10 K markers and 10.0*x* for 3 K markers. At a sufficiently large *x* (10*x* and above), accuracies were comparable between GBS and SNP array data, i.e. 0.48 with 3 K, 0.52 with 10 K, 0.53 with 60 K, and 0.54 with 300 K markers.

**Figure 2:**
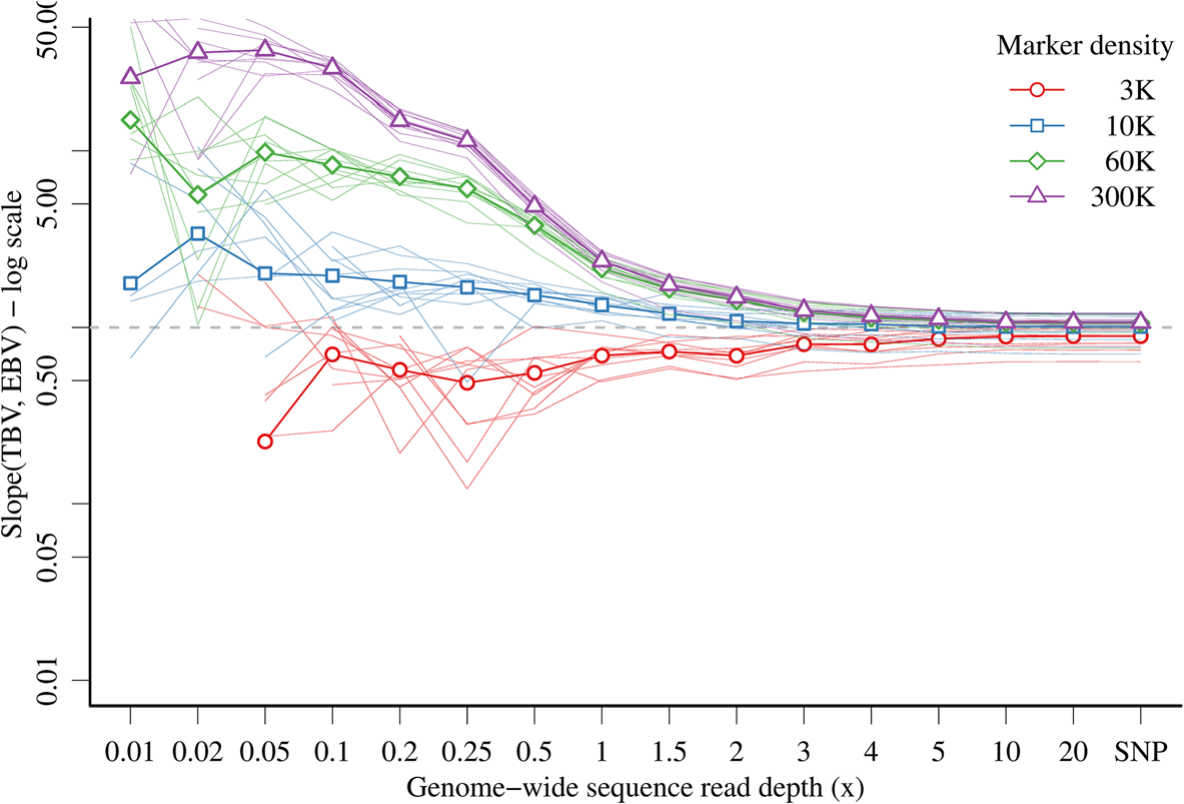
**Bias of genomic prediction with GBS data with the same genome-wide sequence read depth or SNP array data in training and prediction sets and different marker densities (values below 0.01 for 3 K markers and low** ***x*** **were omitted due to the log scale).**

**Figure 3:**
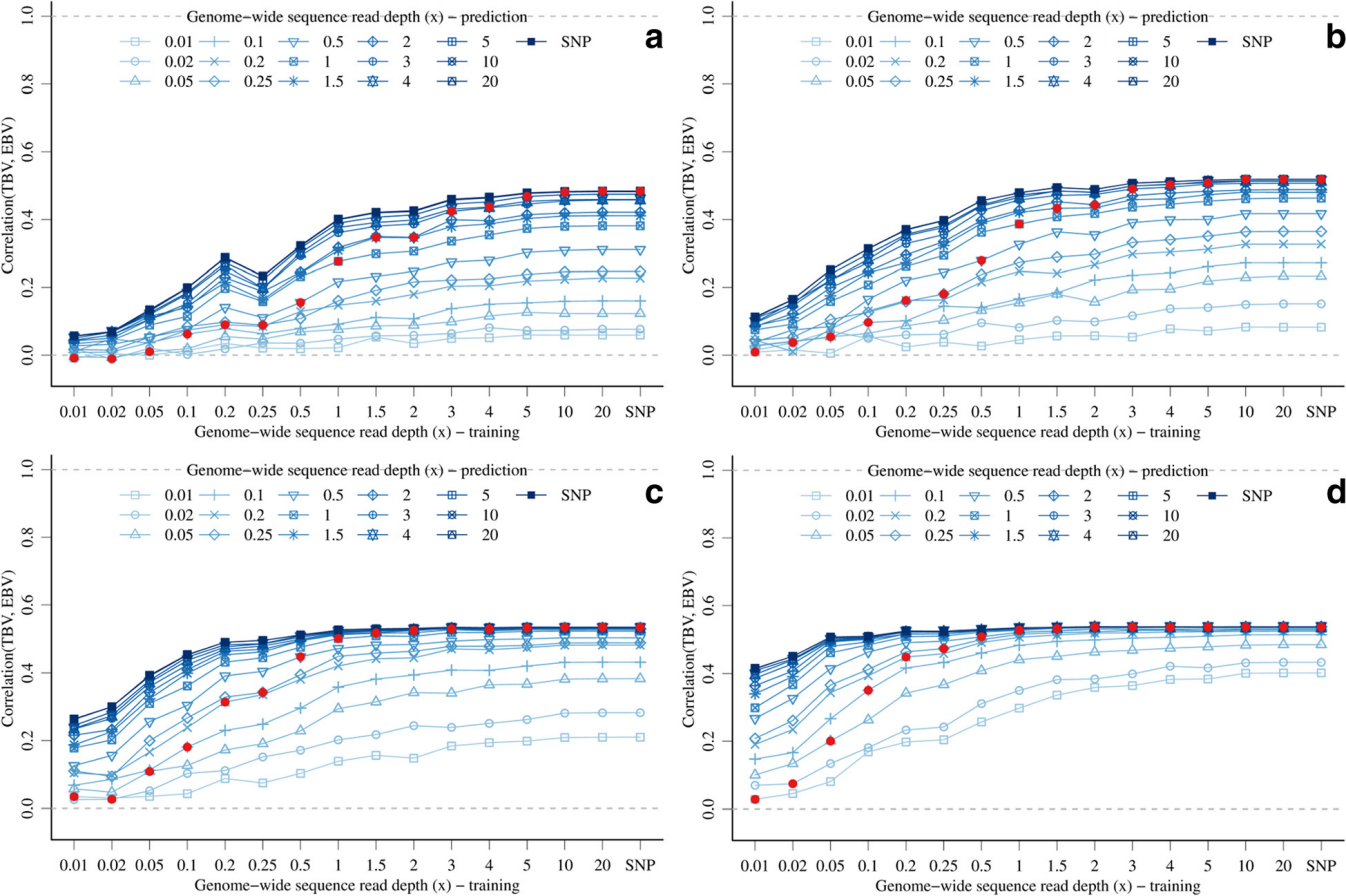
**Accuracy of genomic prediction with GBS data with different genome-wide sequence read depths or SNP array data in training and prediction sets (full red circles show points with equal genome-wide sequence read depth) and different marker densities (a) 3 K top-left, (b) 10 K, top-right, (c) 60 K bottom-left, and (d) 300 K bottom-right.**

Bias of EBV using GBS data was also strongly influenced by *x* and marker density when *x* was the same in the training and prediction sets (Figure 2). In general, EBV were underestimated (bias greater than 1.0) with 10 K, 60 K, or 300 K markers, and overestimated (bias less than 1.0) with 3 K markers. Bias was much greater (as high as 37.1) and highly variable at low *x* and higher marker densities, and decreased with increasing *x.* At a sufficiently large *x* (10*x* and above), biases were comparable between GBS and SNP array data, i.e. 0.89 with 3 K, 1.01 with 10 K, 1.06 with 60 K, and 1.07 with 300 K markers.

(A2) Varying *x* between the training and prediction sets revealed several interactions between *x* and marker density that impacted the accuracy of EBV (Figure 3). Increasing *x* in either the training or the prediction set increased accuracy. When *x* was low in the training set, increasing *x* in the prediction set improved accuracy only a little for low marker densities (e.g., with 3 K markers and 0.1*x* in the training set and 0.1*x* or 1.0*x* in the prediction set, the respective accuracies were equal to 0.06 or 0.11) but accuracy improved progressively more with increasing marker densities (e.g., with 300 K markers and either 0.1*x* in the training set and 0.1*x* or 1.0*x* in the prediction set, accuracies were equal to 0.35 or 0.48). When *x* was intermediate or high (~1.0*x* and above) in the training set, increasing *x* in the prediction set did not improve accuracy for higher marker densities (60 K or 300 K), since the asymptote was largely reached, while there was still some improvement for lower marker densities. Among the tested combinations of *x* in the training and prediction sets, no particular ratio provided substantial benefits, e.g., with 300 K markers, the accuracy was equal to 0.52 with 0.5*x* in the training set and 1.0*x* in the prediction set and also with 1.0*x* in the training set and 0.5*x* in the prediction set (Figure 3d).

**Figure 4:**
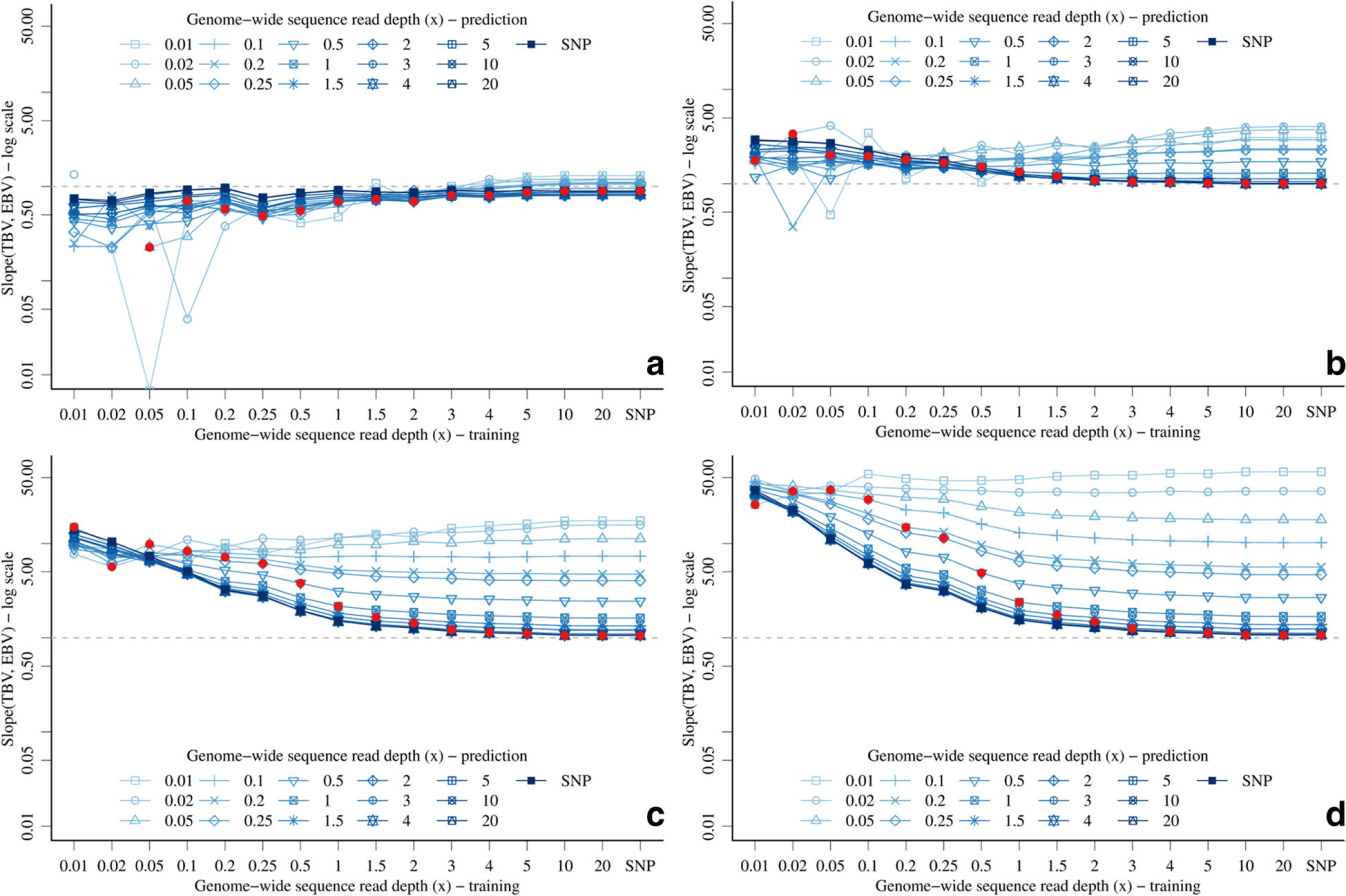
**Bias of genomic prediction with GBS data with different genome-wide sequence read depths or SNP array data in training and prediction sets (full red circles show points with equal genome-wide sequence read depth) and different marker densities (a) 3 K top-left, (b) 10 K, top-right, (c) 60 K bottom-left, and (d) 300 K bottom-right (values below 0.01 for 3 K markers and low** ***x*** **were omitted due to the log scale).**

**Figure 5:**
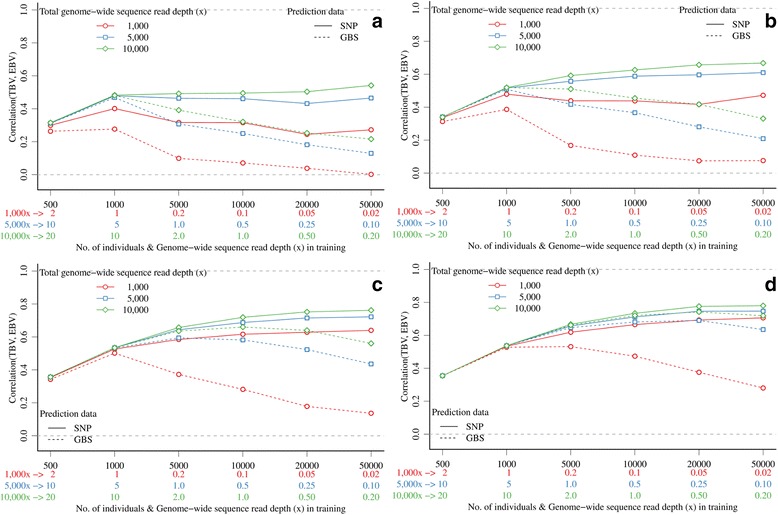
**Accuracy of genomic prediction with GBS data in the expanding training set and SNP array data (solid lines) or GBS data (dashed lines) in the prediction set and different marker densities (a) 3 K top-left, (b) 10 K, top-right, (c) 60 K bottom-left, and (d) 300 K bottom-right.**

There were also many interactions between *x* and marker density in the training and prediction sets for the bias of EBV (Figure 4). Reducing *x* in the training or in the prediction set increased bias. With 3 K markers, the EBV were progressively more overestimated (bias less than 1.0) with a lower *x* in the training set and there was little variation due to *x* in the prediction set (Figure 4a). As marker density increased, EBV were progressively more underestimated (bias greater than 1.0) with a low *x* in the training set and there was much variation due to *x* in the prediction set (Figure 4b, 4c and 4d). When *x* in the training set was low, bias was large and did not vary much for different *x* in the prediction set. With 300 K markers, the effect of *x* on bias was the largest, with bias as high as 57.9 (Figure 4d). However, as *x* increased in the training set, there was a clear interaction with the *x* in prediction: increasing *x* in training reduced underestimation of EBV only if *x* was also increased in the prediction set.

(A3) Keeping the total available *x* constant and varying the number of individuals with GBS data in the training set (and SNP array data for the same markers in the prediction set), accuracies of EBV were generally maximized by using large training sets that comprised individuals with a low *x*, rather than by generating small training sets that comprised individuals with a high *x* (Figure 5). The only exception was with 3 K markers, for which accuracy increased only marginally (with 5000*x* and 10 000*x*) or even decreased (with 1000*x*) when expanding the training set (Figure 5a). With 300 K markers and 10 000*x,* accuracy was only 0.45 with 1000 individuals (10*x* per individual), but was 0.73 with 10 000 individuals (1*x* per individual), 0.78 with 20 000 individuals (0.5*x* per individual), and 0.78 with 50 000 individuals (0.2*x* per individual) (Figure 5d). With GBS data in both the training and prediction sets, there was an optimal combination of *x* and training set size that depended on the number of markers (Figure 5). Accuracies of EBV were generally maximized with high marker densities, large training sets, and large total available *x*, but not beyond a certain *x* per individual. With 300 K markers, asymptotes were reached with training sets that comprised 5000 individuals when using 1000*x* (0.2*x* per individual), 20 000 individuals when using 5000*x* (0.5*x* per individual), or 20 000 individuals when using 10 000*x* (0.25*x* per individual) (Figure 5d). At lower marker densities, asymptotes were reached with much smaller training sets.

**Figure 6:**
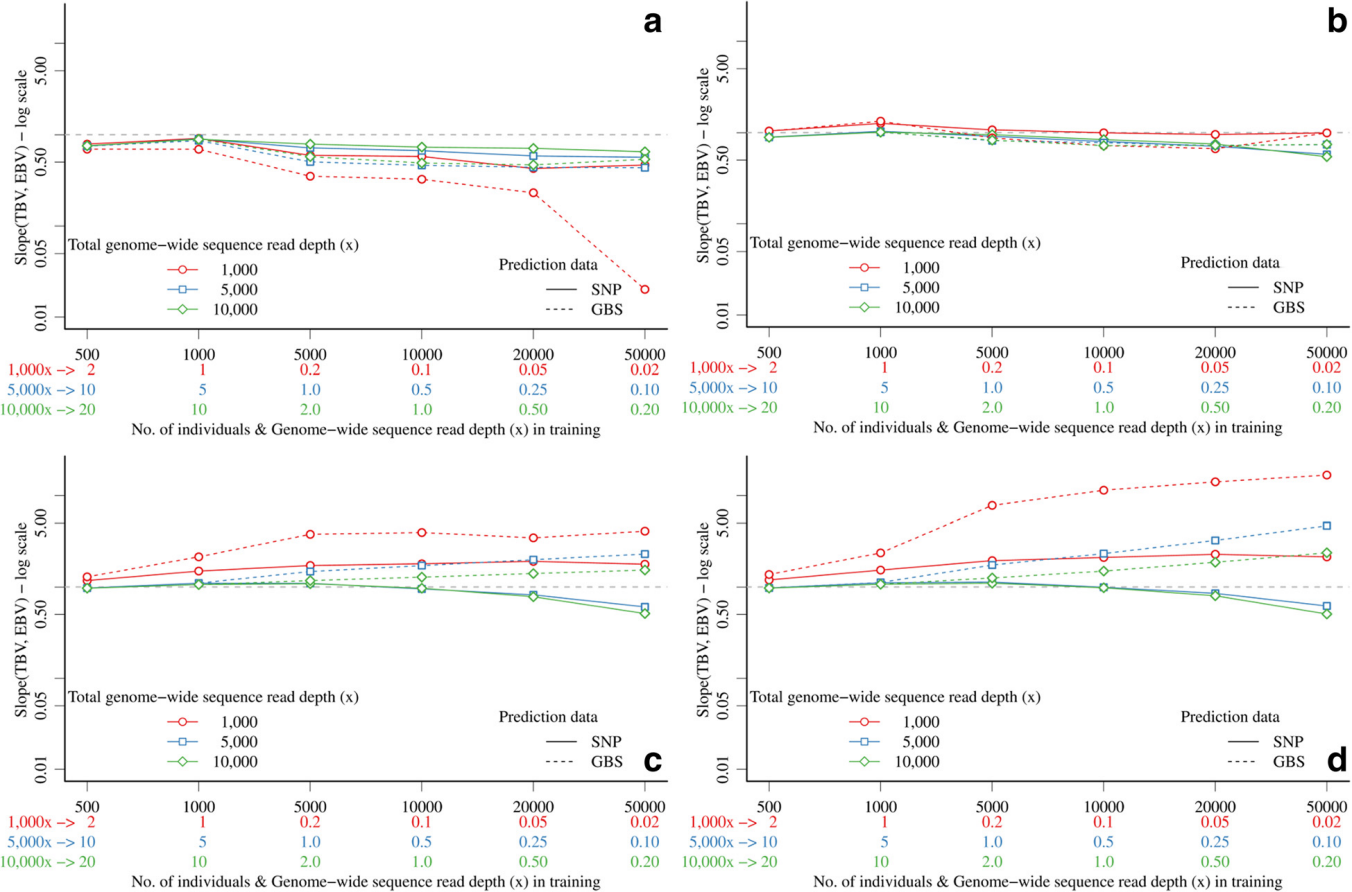
**Bias of genomic prediction with GBS data in the expanding training set and SNP array data (solid lines) or GBS data (dashed lines) in the prediction set and different marker densities (a) 3 K top-left, (b) 10 K, top-right, (c) 60 K bottom-left, and (d) 300 K bottom-right.**

**Figure 7:**
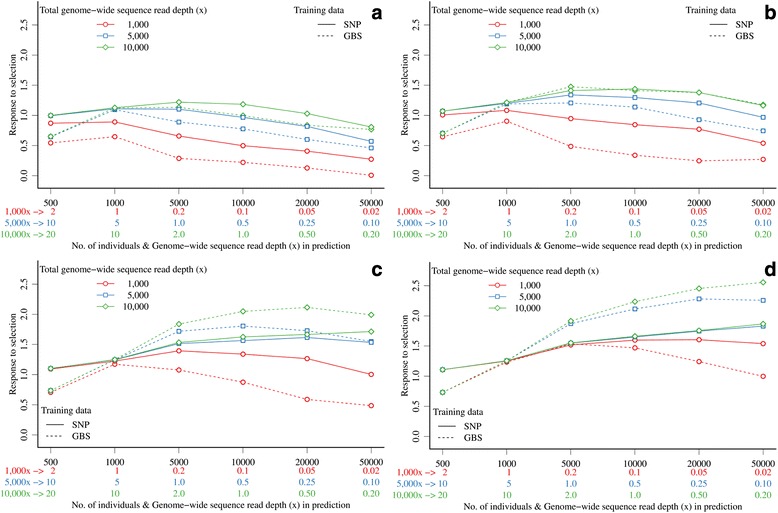
**Response to selection with GBS data in the expanding prediction set based on accuracies of genomic predictions when training on SNP array data with 1000 individuals (solid lines) or when training on GBS data with the same number of individuals and genome-wide sequence read depth as in the prediction set (dashed lines) and different marker densities (a) 3 K top-left, (b) 10 K top-right, (c) 60 K bottom-left, and (d) 300 K bottom-right.**

When a fixed total available *x* was used, so that the number of individuals in the training set could vary, the bias of EBV increased with larger sets of individuals with lower *x* per individual (Figure 6). However, this increase was much smaller than when the training set was constrained to 1000 individuals (Figure 2). This increase was greater with higher marker densities and when prediction was based on GBS data (with the same *x* as the training set) instead of SNP array data.

(A4) For a fixed *x* in the prediction set, the highest response to selection was obtained by generating large prediction sets that comprised individuals with high marker density and low *x* because the high selection intensity compensated for lower accuracy of EBV (Figure 7 and Figure S1 [see Additional file 1: Figure S1]). Small prediction sets that comprised individuals with high marker densities and high quality genotype information led to much lower responses to selection (Figure 7c and Figure 7d). At lower marker densities, the differences in response to selection were smaller or even in favor of higher quality information, since higher selection intensity could not compensate for lower accuracy (Figure 7 and Figure S1 [see Additional file 1: Figure S1]). If 300 K markers were used and 10 000*x* were spread across 500 individuals (20.0*x* per individual), response to selection was equal to 1.11 when training was on SNP array data with 1000 individuals, and equal to 0.73 when training was on GBS data with the same number of individuals and *x* as in the prediction set. Spreading the equivalent of 10 000*x* across 50 000 individuals (0.2*x* per individual) gave a response to selection of 1.87 when training was done on SNP array data and a response to selection of 2.56 when training was done on a larger GBS dataset (Figure 7d).

## Discussion

The results of this study show that GBS can be as accurate as SNP array genotyping for GS in livestock populations and that a high *x* is not necessarily needed to obtain high accuracies. With high marker densities, almost all of the prediction accuracy that can be obtained with high-quality SNP arrays of the same density can be recovered with low *x* (e.g., 0.5*x* to 2.0*x*). Furthermore, NGS approaches provide the user with the opportunity to tailor the quantity and quality of the genotype information to their requirements and some flexibility regarding the number of individuals and genotype quality in both the training and prediction sets. Expanding the training set at the expense of reducing the sequencing depth (and therefore quality of genotyping) for each individual led to higher overall accuracy of EBV. Expanding the prediction set at the expense of reducing the quality of genotyping for each individual led to lower overall accuracy of EBV, but it enabled a higher selection intensity, which in turn resulted in a higher response to selection. Thus, from the perspective of maximizing genetic gain in the next generation, the results suggest that the optimal use of sequencing resources requires an increase in the number of individuals in both the training and prediction sets, at the expense of quality of genotyping. However, at very low *x*, the resulting EBV were biased. This was particularly evident for very low *x* with high marker densities, which conversely are scenarios that, for a fixed amount of financial resources, maximize accuracy and response to selection in the next generation. Biased breeding values may not impact the response to selection in the next generation, but they can impact the long-term response to selection when the value of the breeding value is proportional to the number of genetic contributions that each individual is allowed to make to the next generation.

The finding that low-coverage GBS data can lead to accurate EBV in livestock populations, even in the absence of imputation, is perhaps surprising given that the livestock breeding community has placed major emphasis on ensuring genotype data is accurate. A possible explanation for this finding can be illustrated with the example of 0.1*x* GBS data used to genotype a training population. In this example, at each marker locus approximately 10% of the individuals will have one of their alleles genotyped. The individual and the allele will be sampled at random and this sampling at each marker position will be independent. Thus, the sample of individuals from the population that is used to estimate the phenotypic effect of an allele will be different at each locus. If there is a correlation between alleles at adjacent loci, the sampling of different phenotypes may enable a statistical algorithm to better separate the effects of these loci. Thus, on the one hand, it may be possible to reduce sequencing depth considerably for the construction of very large training populations before the net benefit of assembling larger datasets with low-coverage becomes negative. On the other hand, the quality of genotyping may be much more important for the prediction set. Although reducing the sequencing depth in the prediction set can result in greater selection intensity, it may also be necessary for the genotype of a selection candidate to be much more accurate than that of a training individual. This was clearly observed when marker density was high and with a low *x* in the training set, i.e., prediction accuracy increased greatly when *x* in the prediction set increased. These results are in line with those reported for human populations, which show that low-coverage sequencing (low *x*) of a large number of individuals provides more power for complex trait association studies than deep sequencing (high *x*) of a smaller subset [15,18,19]. The same conclusion was also reached for population genomics studies [28], which showed that it was more advantageous to sequence more individuals at low-coverage than fewer individuals at high-coverage, with an optimum obtained at approximately 1*x*. Extrapolating all these results to whole-genome sequencing for GS suggests that low-coverage sequencing of a large number of individuals could be a viable alternative to deep sequencing of a limited set of “key” individuals of a population [4,29].

A drawback of the low-coverage approach is that heterozygous loci will often be called as homozygous, which limits the use of such data for analyzing the dominance effects. This is not a major limitation for inferring the additive genetic effects, because randomness in calling one or the other allele at a locus provides enough population-wide information to obtain estimates of the allele substitution effects. However, imputation is expected to improve the usefulness of low-coverage data for the analysis of dominance variation by increasing the amount of genotypic information. This information can then be used in the analysis either as discrete genotypes in the case of perfect imputation or as genotype probabilities in the case of imperfect imputation.

This study did not apply imputation algorithms to increase the information content in GBS data, although if it had there could be much less data missing and the effective coverage of each marker could be much higher [18-20]. Increasing the effective coverage could substantially reduce the bias of EBV predicted with low *x* (e.g., 0.5*x* to 2*x*), which, when coupled with high marker densities (i.e., 300 K), would result in accurate EBV and high response to selection in the next generation. In addition, imputation may lead to high levels of accuracy with levels of *x* that are much lower than 0.5*x* to 2*x*, perhaps 0.05*x* to 0.2*x*. From an imputation perspective, properties of GBS data differ from those of SNP arrays that have been commonly used in livestock to date. For example, the density of GBS can be very high, but the information content at each marker position is variable or even missing due to variable *x* along the genome. In addition, some individuals can have mutations in restriction enzyme cut sites, which results in missing genotype calls due to this rather than due to the stochastic nature of the sequencing process. In contrast, SNP array genotypes are called with a high degree of certainty, almost all of the markers that are missing have a certain structure (e.g., imputing from 3 K to 60 K will have the same 57 000 markers missing in all individuals), and the density from which imputation is to be undertaken is much lower (e.g., few hundreds or thousands of markers). For these reasons, the imputation algorithms that have been designed for application in livestock datasets [30-32] are not suited to GBS data. Algorithms that have been designed for applications in human genetics tend to be probabilistic in nature and thus require minor modifications to be applied to GBS data. However, for classical imputation in livestock based on low-density SNP array information, the algorithms that were designed for human datasets are inferior to those specifically designed for livestock datasets, e.g., [32]. Unlike imputation algorithms for human datasets, those for livestock datasets were designed to exploit pedigree information, large family sizes, and abundant close relatives that are prevalent in livestock populations [30-32]. Thus, algorithms for livestock datasets need to be modified to enable imputation of low-coverage sequence data and its large-scale use for GS.

Given that accurate EBV and high response to selection can be obtained with GBS, it represents an attractive alternative to SNP array technology for animal breeders if the cost of generating and using such data is reasonable. Full costs for GBS are difficult to determine and are continually changing with the rapid progress in sequencing technologies and few publications provide a clear breakdown of costs and, in some cases, do not report the full economic impact. Similarly, the cost of SNP array genotyping declines steadily. A recent study on the use of GBS in wheat indicated that future costs per individual would be as low as $10 [9]. However, current full costs of GBS are around $30, which is only one third of the cost of SNP array genotyping for the same number of markers [11]. These values do not include the additional costs of handling GBS data, which, in the absence of computationally efficient and standardized pipelines for livestock data, remains more challenging than that of SNP array data. Two components underlie the costs of low-coverage sequencing: sample preparation and actual sequencing. A recent study on the power of low-coverage sequencing of human genomes [19] indicated that the costs of sample preparation currently range from $15 to $100 per individual and that sequencing costs reach $133 for 1.0*x* sequencing of one individual with a genome size of approximately 3 Gb, which can be assumed to scale linearly with *x* (i.e., 0.1*x* costs $13.3) and the proportion of the genome sequenced. Therefore, GBS-like approaches could be even cheaper since they only sequence a small proportion of the genome. In this study, the total available *x* was spread across different numbers of individuals, which implicitly only includes the actual sequencing costs and thus, assumes that sample library preparation costs are negligible. These assumptions are simplistic, but the purpose of this study was to evaluate the potential of GBS with varying number of individuals, without putting much consideration on costs. Development of imputation algorithms specific to livestock NGS data will substantially reduce sequencing costs per individual and thus the scenarios studied in this work will become realistic.

There are a number of practical limitations to the GBS approach *in lieu* of SNP array genotyping, and simulation studies such as this cannot easily account for these. First, GBS approaches typically sequence the flanking regions of restriction enzyme cut sites, which is equivalent to sequencing subsections of the genome taken at random. While the proportion of the genome sequenced can be tailored through the choice of frequent or rare cutting enzymes, or the use of multiple enzymes, it is a stochastic process and only a proportion of the sequenced sites will contain polymorphic markers. Therefore, to achieve a target marker density, it will be necessary to sequence many more sites, some of which will be uninformative, and this limits the number of samples that can be multiplexed to achieve a target *x*. In addition, the random nature of the sequencing process leads to an uneven *x* across sites and across individuals. Despite equal amounts of input DNA from an individual, there is substantial fluctuation in *x* per individual, with knock-on consequences for genotype quantity and quality per individual [15-17]. However, in spite of these limitations, compared to SNP arrays, GBS has the benefit that its costs of development and of changing the density of the markers are smaller. There are a wide range of suitable restriction enzymes available, which make it possible to sequence different proportions of the genome and thus to vary the density of the resulting data in the population of interest from a few thousand to potentially millions of markers. This could be useful when applying GS in populations with a large effective population size, for example in sheep, goats or beef cattle, for which a large number of markers is needed to achieve sufficiently accurate genomic predictions by capitalizing more on the linkage-disequilibrium information versus the linkage information, e.g., [5]. In addition, unlike the SNPs on arrays, GBS markers do not need to be discovered *a priori* in limited subsets of individuals and therefore do not suffer from the ascertainment bias that affects SNP arrays, e.g., [11-14], and could provide a way to improve across-breed and multi-breed predictions. Coupled with the ability to vary sequencing depth per individual, GBS data has great potential for improving GS.

The impact of sequencing errors was not quantified in this study. Sequencing errors typically occur at 0.5 to 1.0% per raw base and vary somewhat between sequencing approaches [18,33], e.g., for Illumina, the rate of sequencing errors is about 0.1% [33]. Sequencing errors can influence the alignment of reads and genotype calling and thus the downstream analyses. A common approach to improve the accuracy of genotype calls is to use high-coverage sequencing to reduce the effect of sequencing errors [34-36], e.g., with 30*x* the accuracy of genotype calls is more than 99% [34]. However, a large part of the sequencing errors can also be removed from low-coverage data by using sequence data pipelines that trim the ends of sequence reads that tend to have lower quality, filter out individual base reads with low quality, and use probabilistic methods to call genotypes on multiple samples [35]. If low-coverage sequencing is used on relatives, then the shared haplotypes have effectively larger coverage than individual haplotypes, which provides additional information to remove errors and impute missing genotypes [18,19,35].

Applying an error rate of 0.1% to the simulations performed in this study would result on average in 3, 10, 60, and 300 markers with erroneous base reads per individual for marker densities of 3 K, 10 K, 60 K, and 300 K, respectively. These errors would add some additional noise to the genotype calls, which were already quite noisy with low-coverage GBS. Note that at 1*x*, on average all heterozygous loci were called as homozygous, while at 2*x*, on average half of the heterozygous loci were called as homozygous. At 0.5*x*, on average half of the loci were not called at all and the other half had all heterozygous loci called as homozygous. With 0.1% erroneous base reads, the amount of wrong genotype calls would not increase drastically and would therefore not invalidate the main conclusions of this study. A more efficient approach than calling genotypes would be to calculate genotype probabilities conditionally on the observed sequence reads from each individual and its relatives and sequence error rates [18,19,35]; this should be further studied in the future. Another consequence of sequencing errors is that they increase the uncertainty of inferred genotype calls or genotype probabilities, which in turn reduces the signal from the data. However, due to the largely underdetermined systems with more correlated markers than phenotyped individuals that underpin GS, it is essential to increase the number of genotyped and phenotyped individuals, even at the expense of a lower quality of genomic information. Low-coverage sequencing approaches such as GBS provide a way to manage these aspects such that the genetic gain in a population can be maximized.

## Conclusions

In conclusion, NGS techniques used for genotyping such as GBS have potential advantages for genomic selection in livestock. Our results show that genomic prediction using unimputed GBS data gives comparable accuracies to using SNP array data with the same number of markers, even if the genome-wide sequence read depth (*x*) is as low as ~1*x* and large numbers of markers are available. The ability to vary the quality of genotyping per individual (by varying the sequencing effort) makes it possible to reduce the cost of genotyping of a large number of individuals and therefore to increase the accuracy of prediction and selection intensity. Similar strategies could be developed for low-coverage sequencing of whole genomes. Further developments in sequencing and imputation techniques are necessary to improve the cost effectiveness of such strategies for their application to real populations.

## Additional files

Additional file 1: Figure S1. Accuracy of genomic prediction with GBS data in the expanding prediction set when training on SNP array data with 1000 individuals (solid lines) or when training on GBS data with the same number of individuals and genome-wide sequence read depth as in the prediction set (dashed lines) and different marker densities (a) 3 K top-left, (b) 10 K, top-right, (c) 60 K bottom-left, and (d) 300 K bottom-right. Accuracy of genomic prediction with GBS data in the expanding prediction set.
