# Supplementary material for: Potential of genotyping-by-sequencing for genomic selection in livestock populations
Source: Genet Sel Evol. 2015 Mar 1;47(1):12. doi: 10.1186/s12711-015-0102-z (PMC4344748; doi:10.1186/s12711-015-0102-z)
Supplement: Additional file 1: Figure S1. — Accuracy of genomic prediction with GBS data in the expanding prediction set when training on SNP array data with 1000 individuals (solid lines) or when training on GBS data with the same number of individuals and genome-wide sequence read depth as in the prediction set (dashed lines) and different marker densities (a) 3 K top-left, (b) 10 K, top-right, (c) 60 K bottom-left, and (d) 300 K bottom-right. Accuracy of genomic prediction with GBS data in the expanding prediction set. [file 12711_2015_102_MOESM1_ESM.pdf]

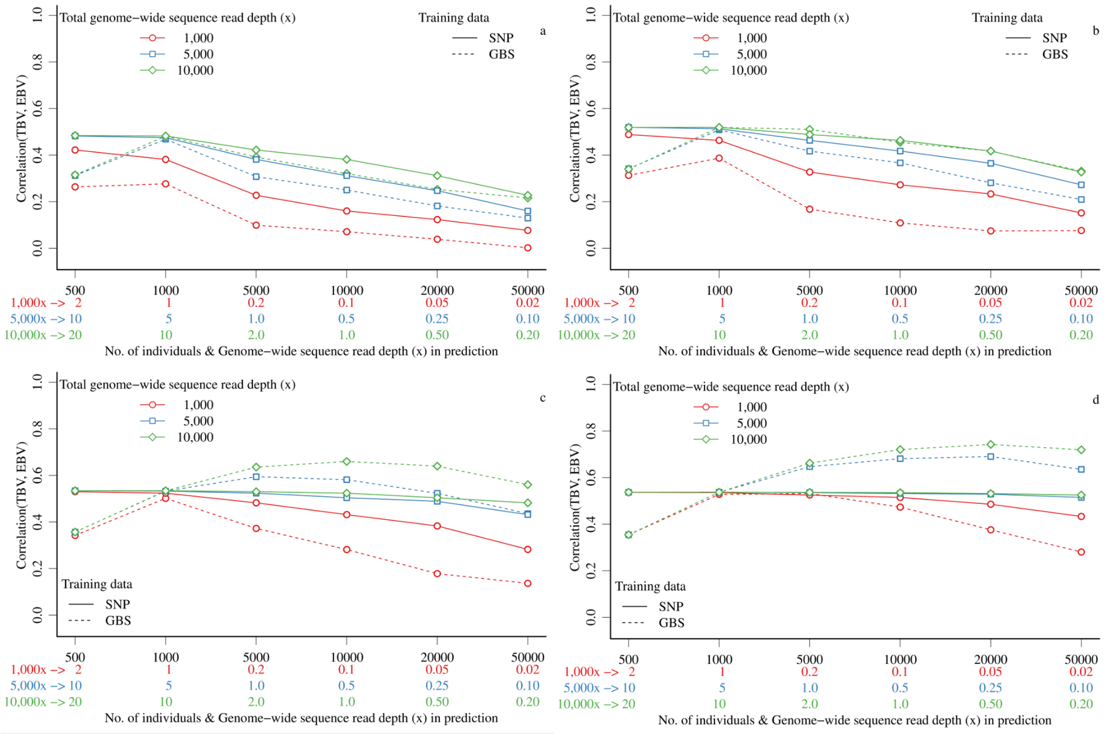

Figure S1. Accuracy with GBS data in the expanding prediction set and SNP array data (solid lines) or GBS data (dashed lines) in training. Training on SNP array data involved 1,000 individuals, while training on GBS data involved the same number of individuals and genome-wide sequence read depth as in the prediction set. Different marker densities are shown in top-left – a) 3K, top-right – b) 10K, bottom-left – c) 60K, and bottom-right – d) 300K.
